# A Cooperative Machine Learning Approach for Pedestrian Navigation in Indoor IoT

**DOI:** 10.3390/s19214609

**Published:** 2019-10-23

**Authors:** Marzieh Jalal Abadi, Luca Luceri, Mahbub Hassan, Chun Tung Chou, Monica Nicoli

**Affiliations:** 1 School of Electrical Engineering, Sharif University of Technology, Tehran PO Box 11365-11155, Iran; 2 School of Computer Science and Engineering, University of New South Wales, Sydney, NSW 2052, Australia; Mahbub.Hassan@unsw.edu.au (M.H.); ctchou@cse.unsw.edu.au (C.T.C.); 3 Istituto Sistemi Informativi e Networking, University of Applied Sciences and Arts of Southern Switzerland, 6928 Manno, Switzerland; luca.luceri@supsi.ch; 4 Dipartimento di Ingegneria Gestionale (DIG), Politecnico di Milano, 20133 Milano, Italy; monica.nicoli@polimi.it

**Keywords:** IoT, smart environments, context aware application, machine learning, indoor localization

## Abstract

This paper presents a system based on pedestrian dead reckoning (PDR) for localization of networked mobile users, which relies only on sensors embedded in the devices and device- to-device connectivity. The user trajectory is reconstructed by measuring step by step the user displacements. Though step length can be estimated rather accurately, heading evaluation is extremely problematic in indoor environments. Magnetometer is typically used, however measurements are strongly perturbed. To improve the location accuracy, this paper proposes a novel cooperative system to estimate the direction of motion based on a machine learning approach for perturbation detection and filtering, combined with a consensus algorithm for performance augmentation by cooperative data fusion at multiple devices. A first algorithm filters out perturbed magnetometer measurements based on a-priori information on the Earth’s magnetic field. A second algorithm aggregates groups of users walking in the same direction, while a third one combines the measurements of the aggregated users in a distributed way to extract a more accurate heading estimate. To the best of our knowledge, this is the first approach that combines machine learning with consensus algorithms for cooperative PDR. Compared to other methods in the literature, the method has the advantage of being infrastructure-free, fully distributed and robust to sensor failures thanks to the pre-filtering of perturbed measurements. Extensive indoor experiments show that the heading error is highly reduced by the proposed approach thus leading to noticeable enhancements in localization performance.

## 1. Introduction

As technology advances, computers and mobile devices are becoming the leading digital platforms in our everyday life. Currently, ubiquitous computing is an emerging field that infers the contextual information of the environment from connected smart devices [1,2]. As the use and reliance on smart devices grows within public domain, context aware technologies are expected to become an essential requirement in the development of smart cities. In this sense, the location of people is an important source of contextual information and a key enabler of novel location-based services (LBS) [3,4,5].

Global Navigation Satellite Systems (GNSS) are the most widely used positioning solutions [6], however, they can be easily blocked in indoor environments as they cannot handle the high attenuation and sever multi-path effects that occur indoors. This calls the development of new solutions for indoor localization. Network-based localization systems are proposed as a valuable substitute, but conventional techniques mostly require expensive ad-hoc infrastructures, which may not always be available [7,8,9,10].

Recently, new promising solutions have been emerging in the context of Internet of Things (IoT), where sophisticated sensing, computing and networking technologies are used to interconnect large numbers of smart devices and let them cooperate for monitoring and control goals, included location sensing [11,12,13,14]. This opens the door to infrastructure-free localization solutions such as Pedestrian Dead Reckoning (PDR), which exploits information from inertial sensors embedded in users devices [15,16]. The basic principle of PDR is to estimate the user displacements per step [17]: the position is recursively obtained by combining the last known position with the step length and heading measured by inertial sensors. While step length can be assessed rather accurately [18], the estimation of heading in indoor environments is extremely challenging. Gyroscope and magnetometer are usually employed to address this issue, however, the gyroscope can be used for a short time as it seemingly accumulates error over time [19], whereas magnetometer gets perturbed from man-made infrastructures [20]. Therefore, accurate heading estimation remains an open issue in PDR-based indoor localization [21,22].

Although an individual magnetic sensor may experience perturbation, multiple pedestrians walking in the same direction could in principle collaborate to overcome the effect of isolated perturbations. Emerging device-to-device (D2D) communication standards, such as WiFi-Direct [23], could be readily used to share heading information based on magnetometer measurements in real time between different pedestrians. The implementation of such collaboration, however, will have to address three major challenges: (1) how to detect pedestrians who are walking in the same direction, (2) how to establish whether a heading estimate is severely affected by magnetic perturbation, and (3) how to fuse data from many pedestrians scattered over space without a central control and taking into account the different degree of reliability of the collected data.

In this paper, a new PDR-based localization for an IoT-enabled smart-cities’ paradigm is proposed. We assume the mobile users carry smart devices (smart phones in the experimental tests), which are equipped with inertial sensors and they are interconnected via WiFi-Direct. We propose a novel distributed and collaborative PDR system based on cooperative machine learning (CML) that enhances magnetic sensors embedded in the smart phones and accurately estimates the heading of pedestrians that are closely located. Performance analysis indicates an error reduction of 86% in heading estimation of pedestrians that leads to substantial improvement of 79% in the accuracy of pedestrian localization using conventional PDR for indoor environment.

### 1.1. Related Works

Indoor navigation is highly challenging due to the complex propagation conditions, the density of objects or installation of equipment and infrastructure. The proposed algorithms for Indoor Positioning System (IPS) can be categorized into two groups: network-based and non-network-based systems. The former systems rely on network and infrastructure and need reference points (e.g., access points) for identifying user locations and the latter rely on inertial sensors and PDR algorithms.

Network-based systems utilize multi-lateration/angulation and fingerprinting techniques to localize the user [7,8,24]. Generally, radio frequency (RF) beacon tags such as ultra wideband (UWB) [25], acoustic [26], Bluetooth [27] or radio frequency identification (RFID) [28,29] are adopted for multi-lateration/angulation and measure the times of arrival (TOA) or the angle of arrival (AOA) to approximate the position of the user. Alternately, fingerprinting techniques make use of received signal strength (RSS) of WiFi [30], cellular signals [31], geomagnetic field measurements [32] or ambient measurements [33] to localize the position of the user. The localization accuracy of network-based IPSs mostly depends on the physical environment, the positioning algorithm, the number and geometrical arrangement of reference points [34,35]. A recent study exploits the fingerprint database of patterns formed by magnetic flux intensity values and deep learning algorithm to solve the need of updating the database periodically [36]. Although they generally achieve acceptable performance, they require an expensive infrastructure which may not be available, as in emergency situations.

Non-network-based systems are usually based on PDR algorithms and inertial sensors. Unlike network-based localization systems, they do not require any pre-arranged infrastructure, and the user’s location estimate is updated sequentially at each step [17]. PDR systems commonly use gyroscope to measure changes in heading, but the location estimate of these systems are extensively noisy and the performance highly degrades over time [37]. The improved heuristic drift reduction (iHDR) is an approach that estimates the drift component to reduce the heading error [38]. This system can be sensitive to accelerations and its performance can be deteriorated in complex buildings. Despite indoor magnetic field is highly perturbed, the authors in [39,40] combine heading estimates from magnetometer and gyroscope and implement it over a PDR framework with an extended Kalman filter. These multi-sensor fusion based approaches provide beacon-free PDR, however their performance can degrade for long distance in indoor environment.

As PDR alone is not a promising solution in complex indoor environments, hybrid and collaborative solutions have been proposed to improve the performance of IPS. The hybrid solutions rely on the combination of several technologies to localize a user [41,42], whereas collaborative methods exploit the location estimates of multiple users and cooperate between them to improve the localization performance [43,44,45,46,47]. For instance the combinations of Wi-Fi, magnetic field and RFID in the fingerprinting approach [41], PDR with iBeacon [42], and PDR with UWB [48] are some possible hybrid solutions. People-centric navigation (PCN) [43] is an example of cooperative approaches that utilizes the feature of “group activity” measuring the mobile phone’s acceleration, heading and Bluetooth RSS and detects similarity among subjects to correct PDR traces of group members. The authors in [44] propose a smart phone-based collaborative approach, where devices of pedestrians physically close to each other use proximity information to improve their PDR location estimates. Cooperative approaches have been investigated also in infrastructure based systems (i.e., relying on a set of anchors), by exploiting factor graphs and message passing algorithms [45]. A distributed approach based on the consensus algorithm is proposed in [46] for both fixed and mobile users.

### 1.2. Original Contributions

Due to the highly localized nature of indoor magnetic anomalies, multiple pedestrians walking in the same direction but stepping on different locations are likely to experience different level of magnetic disturbance. Despite an individual magnetic sensor may experience some perturbations, pedestrians who are walking in the same direction should have similar heading measurements, therefore we can combine their heading estimates to overcome the effect of isolated perturbations and improve the heading accuracy.

The proposed cooperative machine learning PDR (CML-PDR) system consists of three main components: Magnetic Perturbation Detection (MPD), Pedestrian Clustering (PC) and Distributed Data Fusion (DDF). A machine learning algorithm is first designed for MPD to detect whether a heading estimate is perturbed by magnetic anomalies or not. Only estimates within a selected error margin are retained and used for heading estimation. The second component, PC uses a machine learning algorithm to detect groups of users heading in the same direction based on Received Signal Strength (RSS) measurements exchanged over D2D links. Finally DDF combines the retained heading measurements from users in the same group in a fully distributed manner by a consensus algorithm. We assess the performance of CML by an extensive experimental campaign carried out in complex localization scenarios, for varying configurations of user density and network connectivity.

To the best of our knowledge, this is the first approach that combines machine learning with consensus algorithms for cooperative PDR. With respect to other infrastructure-free methods [43,44] that exploit the similarity in groups of people moving together to improve the localization performance, here the proposed approach is fully distributed (whereas [44] relies on a centralized server) and can aggregate the information of any number of connected devices thanks to the consensus approach (while in [43] the fusion is between two users per time). The proposed approach is thus robust (thanks to the prefiltering of perturbed sensor data), it scales well with the network dimension and is not sensitive to a single point of failure (i.e., the server). A preliminary version of this work was presented in [49]. Here the work is extended by including the pedestrian clustering component, the study of the localization performance for varying number of users and connectivity degree, and extensive experimental tests to validate the proposed method.

To summarize, our contributions are as follows:

- We propose a machine learning algorithm to detect whether magnetometer measurements collected by the device-embedded sensors are severely perturbed. The algorithm is completely self-sufficient, i.e., it detects perturbed measurements using only features extracted from magnetic readings. We show that this algorithm can detect perturbation with an accuracy of 95%.
- We design an algorithm to detect pedestrians walking in the same direction, using a machine learning approach. The algorithm exploits RSS data extracted from WiFi beacons exchanged by pedestrians’ mobile devices. Our experiments indicate that the proposed algorithm can detect pedestrians walking in the same direction with an accuracy of 90%.
- We prove that fusion of heading data can be carried out in a fully distributed way, with negligible performance loss compared to the centralized fusion. Different consensus approaches are considered for data fusion. The performances of the algorithms are analyzed in terms of localization accuracy and convergence rate, for varying number of users and connectivity graphs.
- We evaluate the performance of the proposed architecture utilizing experimental data. We find that the proposed architecture achieves an error reduction of 86% in the heading estimation and 79% in user localization compared to PDR legacy architecture. In order to show the reliability of the proposed approach, we evaluated the performance for different experiments at different locations. Our result indicates that the error reduction is consistent for different settings and independent of the location and time of the experiments.

The paper is organized as follows. We first introduce the CML-PDR system in Section 2 followed by the experiments carried out for evaluation in Section 3. We then discuss the three main components of the proposed architecture in detail in Section 4, Section 5 and Section 6. Finally, in Section 7 we evaluate the performance of the system and we draw the main conclusions in Section 8.

## 2. Cooperative Machine Learning PDR

**Figure 1:**
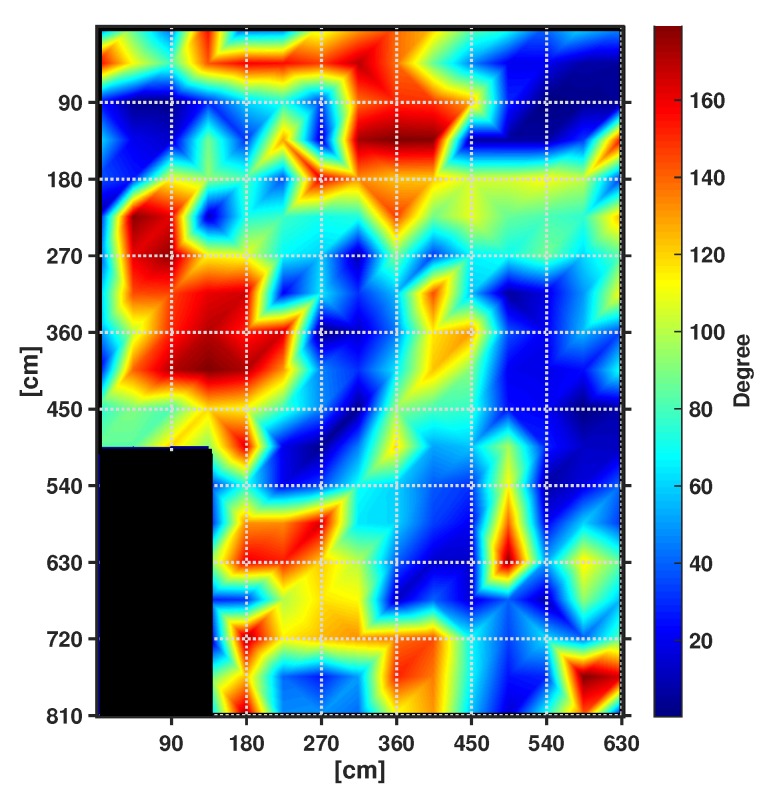
Heading Error Map. Blue color indicates locations with low level of perturbation, while red color represents points highly perturbed. The black color at the bottom left corner identifies a space outside the room.

We introduce a PDR system that utilizes only sensors embedded in the user device (e.g., a smart phone or any IoT device) and D2D connectivity between pedestrians. D2D communications are used for data fusion between multiple pedestrians to take benefit from the spatial diversity of magnetic measurements in indoor environment. Given that the Earth’s magnetic field value is stationary and known in any given spot of the world [50,51], in our previous study we have demonstrated that indoor magnetic perturbation is highly localized in space [49]. As an example, Figure 1 shows a heat map of the heading estimate error observed at 264 locations arranged in a grid in an indoor space at the University of New South Wales (UNSW). During the experiment, a Samsung Galaxy SIII phone was placed so that it has a heading of $h=280^{\circ }$. Apparently, numerical results indicate that in the surrounding of a given position, only a limited area are severely perturbed. Therefore, if closely-located users head the same direction, then only some of their magnetometers are likely to be perturbed. This means that, although a single pedestrian may not be able to rely on his/her heading, he/she can use the estimates of other pedestrians walking in the same direction that are not experiencing perturbation.

**Figure 2:**
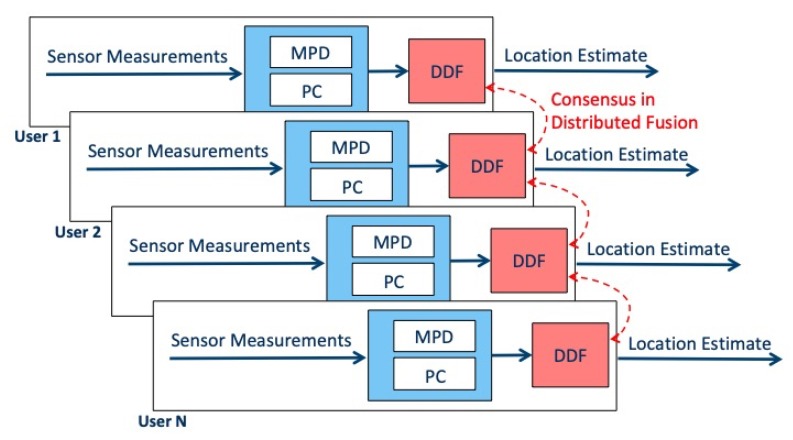
Cooperative Machine Learning PDR Architecture.

The CML-PDR system architecture is displayed in Figure 2 and each component is described below:

1. The MPD component detects highly corrupted magnetometer readings based on a ML approach. Perturbed measurements have to be excluded from data fusion to avoid large positioning errors. For this reason, only heading readings that are believed to fall within a selected error margin are retained and passed to the subsequent processing steps.
2. The PC component identifies groups of pedestrians walking in the same direction. This detection has a key role as it aggregates users that can benefit from measurements fusion. ML approach is used to perform this clustering, based on RSS measurements exchanged among users.
3. The DDF component aggregates the heading measurements that have been filtered by MPD and PC in a fully distributed way. The outcome is shared with all the users belonging to the same group, including those that were excluded from data fusion.

## 3. Experiments

We used two different sets of experiments to analyze the performance of CML-PDR referred to as the Small- and Large-Scale Scenarios.

**Figure 3:**
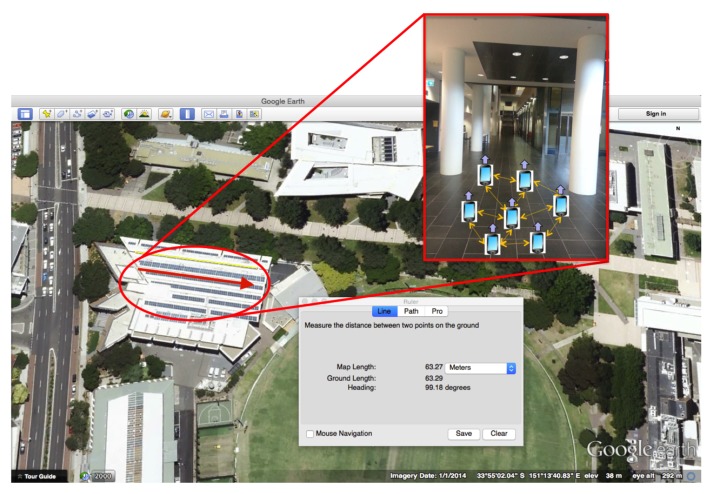
An indoor localization scenario with multiple pedestrians walking in the same direction along a corridor. Each smart phone in the figure denotes a pedestrian, while the edges connecting the devices indicate D2D connections between users sharing the heading. The figure also shows the satellite image (obtained from Google Earth) of the Tyree Buildings at the University of New South Wales, which was one of the building utilized in the experimental campaign.

The Small-Scale Scenario was carried out to validate the individual system components and the overall localization performance for different user configurations, i.e., with pedestrians walking in the same direction, crossing or turning and increased complexity of trajectories. A small group of users (up to 4 pedestrians) in the validation was considered. The experiments of Small-Scale Scenario were conducted in indoor environment in Tyree Building at UNSW in Sydney, Australia. As represented in Figure 3, for each experiment, multiple pedestrians were walking in the same direction along a corridor. Each smart phone in Figure 3 denotes a pedestrian, while the edges connecting the devices indicate D2D connections between users sharing the heading. Figure 3 also shows a satellite image of Tyree Building utilized in the experimental campaign obtained from Google Earth, from which we obtain the heading ground truth values.

The Large-Scale Scenario is considered (i) to verify the impact of the degree of cooperation among users on the localization accuracy and (ii) to test the performance of CML-PDR with a larger group of pedestrians. For this second study, we focused on a larger set of users (up to 18 pedestrians) walking in a rigid formation over different buildings at UNSW with varying degree of spatial diversity, and we considered different radius of cooperation for distributed data fusion. The two measurement campaigns are described in the following.

### 3.1. Small-Scale Scenario

This campaign involved four pedestrians (two males and two females) walking in a corridor of the Tyree Building at UNSW.

Two different corridors were considered, one in the lower ground (Corridor LG) and the other at the ground level (Corridor G) of the Tyree Building. The two corridors have the same dimension (27 m long and 4 m wide) and orientation ($99.18^{\circ }/279.18^{\circ }$). The corridor LG and the location of the Tyree Building are shown in Figure 3.

The pedestrians involved in the campaign are referred to as U1, U2, U3 and U4. Pedestrian U4 carried a phone configured as a WiFi-Direct server transmitting WiFi beacons every 0.3 s. In this campaign, we used 2 different apps to collect RSS and magnetometers measurements and it was not possible to run both apps simultaneously on a smartphone. Therefore we preferred to pursue the data collection with two different smartphones. Every user carried one phone in each hand, one to collect magnetometer data at frequency of 100 Hz and the other to measure RSS of the beacons transmitted by the WiFi-server. The former phone was held horizontally in front of the user so as to align the phone heading with the walking heading, while the latter was carried in a natural swinging mode. The pedestrians were instructed to walk together with the same speed. All the smartphones in our data collection campaign were set to a unique time and date that helps us to synchronize the collected data between apps and users.

We carried out the experiments in three different topologies A, B and C. They are described as follows and detailed in Table 1. In topology A, all the pedestrians shared the same direction. In topology B, pedestrian U4 walked in the opposite direction with respect to the other three users. Topology C is similar to topology B except that U1 walked in the opposite direction with respect to the other three users. Therefore, in topology A we had a group of four pedestrians heading in the same direction, while in topologies B and C we had a group of three pedestrians sharing their direction. We also considered the two distinct orientations ($99.18^{\circ }$ and $279.18^{\circ }$) for each type of topology.

### 3.2. Large-Scale Scenario

**Figure 4:**
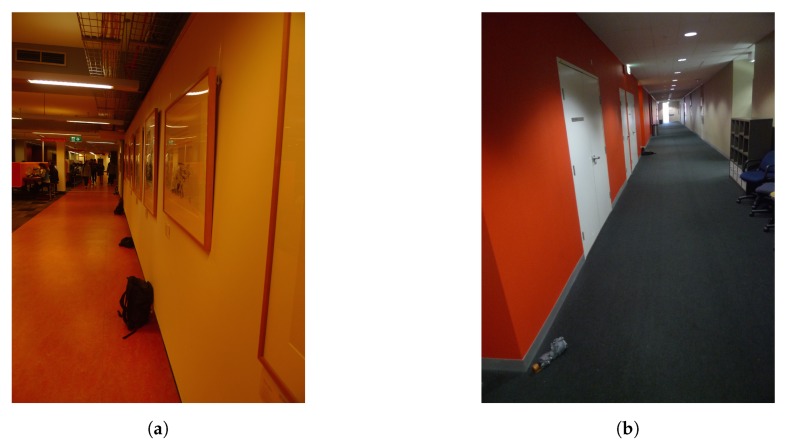
Library and Robert Webster Building Corridors. (**a**) Library Corridor; (**b**) Robert Webster Corridor.

Further experiments were carried out in multiple buildings at UNSW in order to ensure diversity in environmental conditions, especially with respect to magnetic perturbations. As detailed in Table 2, different corridors and orientations were chosen, and we use this set of data to test the performance of CML-PDR with a larger cluster of pedestrians and for different degrees of cooperation among users. Figure 4 shows the corridors of Library and Robert Webster Building.

**Figure 5:**
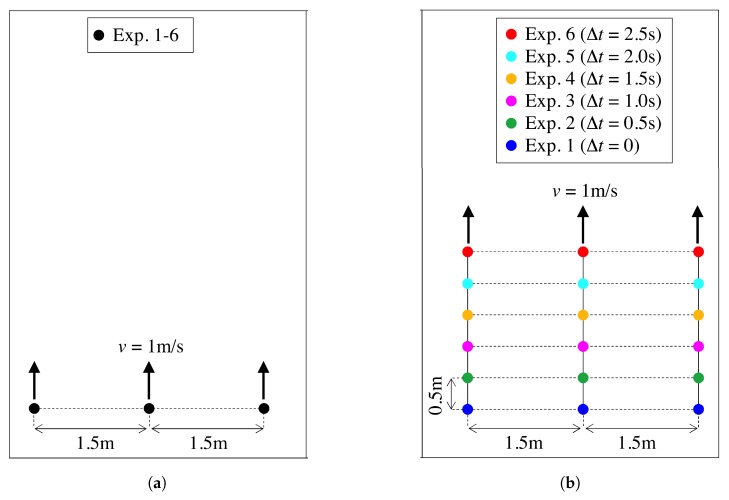
Starting user position in the experiment (3 users) and in the enlarged scenario (18 users), respectively. The extended dataset is obtained by aggregating the data of the 6 experiments with a relative time offset (as indicated in the legend) to form a regular 3 × 6 user grid. (**a**) Experiment Setting (3 Users); (**b**) Large-Scale Scenario (18 Users).

As it can be observed in Table 2, a set of 6 experiments per building was performed, except for Scenario ID 5 where only 5 experiments were carried out. For all the experiments, 3 subjects walked along the corridor, aligned in the traversal direction with inter-distance of 1.5 m, taking a step of 0.5 m every 0.5 s (i.e., with constant speed of 1 m/s), as depicted in Figure 5a. Each subject held either a HTC1X or HTC1X+ device, that collected magnetometer measurements at the rate of 16 Hz.

Figure 5a shows the original user configuration in each experiment, where users followed the same trajectories in each experiments. From this set of experiments, we recreated an enlarged cluster of pedestrians by combining the offline data of multiple experiments in each building. More specifically, for every building of Table 2, we aggregated the measurements collected in the 5 (for scenario ID 5) or 6 (for all the other scenarios) experiments for 3 users, to get a virtual extension to K=15 or K=18 users, respectively. Thereby, as depicted in Figure 5b, to get the cluster of K=18 users, we simply aggregated the data of 6 experiments with a time shift of 0.5 s, so as to obtain a regular grid of 6×3 users, with vertical spacing 0.5 m (in the walking direction) and horizontal spacing of 1.5 m (in the traversal direction).

## 4. Magnetic Perturbation Detection (MPD)

In this section we develop a method to be used at each user device to detect unreliable heading estimates to exclude them from data fusion.

The detection of unreliable heading measurements is performed by each user device at time step *t* using the measurements of magnetometer. These measurements are in the device coordinate system, {x,y,z}, that is known as local coordinate in the literature. We refer these measurements as raw data and denote them by $\left\{{\tilde{m}}_{x}\left(t\right),{\tilde{m}}_{y}\left(t\right),{\tilde{m}}_{z}\left(t\right)\right\}$.

In order to calculate the heading, we need the magnetometer measurements in the Earth coordinate system, therefore the raw data from the smart phone coordinate system should be converted to the Earth coordinate system using transformation matrix [52]. There is no need to convert the device coordinate system to the Earth coordinate system if the device and the Earth coordinate system are aligned. Considering the fact that the Earth coordinate system is horizontal, we asked the users to hold the device horizontally during data collection for all the experiments to avoid the conversion between the device and the Earth coordinate systems. For the device held horizontally, the instantaneous heading of the considered subject is computed as [53]:

(1) $$\tilde{h}\left(t\right)=\tan^{-1}\left(\frac{{\tilde{m}}_{y}\left(t\right)}{{\tilde{m}}_{x}\left(t\right)}\right).$$

The Earth’s magnetic field is stationary and known in any given area [50,51], however the value of $\left\{{\tilde{m}}_{x}\left(t\right),{\tilde{m}}_{y}\left(t\right),{\tilde{m}}_{z}\left(t\right)\right\}$ at a specific location changes depending on the orientation of the device. In order to provide some hints to the machine learning algorithm to detect erroneous heading error, we utilize the International Geomagnetic Reference Field (IGRF) [54] for Sydney as a reference. Each IGRF datum is a triplet (H,F,I), where *H* and *F* are respectively, the horizontal and total magnetic field, and *I* is the inclination angle of the Earth magnetic field in the given location. These three quantities are scalar values and related to the magnetic field strength $\left\{{\tilde{m}}_{x}\left(t\right),{\tilde{m}}_{y}\left(t\right),{\tilde{m}}_{z}\left(t\right)\right\}$ at each point by:

(2) $$\begin{array}{cc} \tilde{F}\left(t\right) & =\sqrt{{\tilde{m}}_{x}{\left(t\right)}^{2}+{\tilde{m}}_{y}{\left(t\right)}^{2}+{\tilde{m}}_{z}{\left(t\right)}^{2}}\\ \tilde{H}\left(t\right) & =\sqrt{{\tilde{m}}_{x}{\left(t\right)}^{2}+{\tilde{m}}_{y}{\left(t\right)}^{2}}\\ \tilde{I}\left(t\right) & =\tan^{-1}\left(\frac{{\tilde{m}}_{z}\left(t\right)}{\tilde{H}\left(t\right)}\right). \end{array}$$

Since the reference IGRF is known at Sydney as the reference location, we computed the deviation to the reference values and obtained the error in the IGRF components as: $F_{err}\left(t\right)=\tilde{F}\left(t\right)-F,H_{err}\left(t\right)=\tilde{H}\left(t\right)-H$, and $I_{err}\left(t\right)=\tilde{I}\left(t\right)-I$. We append these error figures to the raw magnetic field measurements to obtain a vector $M\left(t\right)=[{\tilde{m}}_{x}\left(t\right),{\tilde{m}}_{y}\left(t\right),{\tilde{m}}_{z}\left(t\right),F_{err}\left(t\right),H_{err}\left(t\right),I_{err}\left(t\right)]$, named extended magnetic field measurement vector.

Since the magnetometer readings are affected by random error, the instantaneous heading estimate fluctuates over time. Therefore we computed the mean of heading estimate $\bar{h}$ by averaging the instantaneous headings within a time window. A time window of T=0.6 s is selected as this is roughly the time a pedestrian takes to make one step [18]. Let $h_{\mathrm{true}}$ be the true heading, then $e=|\bar{h}-h_{\mathrm{true}}|$ is the absolute heading error. For a given threshold γ, we consider a mean heading $\bar{h}$ without perturbation if e≤γ, otherwise we consider it as perturbed. Formally we define two classes $C_{0}$ and $C_{1}$, where the class $C_{1}$ (resp. $C_{0}$) contains all mean headings that are not perturbed (perturbed). In our previous study [49], we show that there is an optimum threshold that minimizes the estimated heading error. We evaluated several values of the threshold γ for all the experiments in our dataset and selected $\gamma =10^{\circ }$ as the optimum threshold.

In order to build a model that detects whether the heading measurement is corrupted or not, we first need to create our dataset. Based on our previous study [49], statistical mean was chosen as the selected feature for our data. We have also used correlation based feature selection [55] to select the best features and the results show that the three magnetic components are fundamental features of MPD. Therefore, we first collected all the extended magnetic field measurement vectors and then calculated the mean vector $\bar{M}=E\left[M\left(t\right)\right]$ by averaging the samples over the considered time window where T=0.6 s. For each time window, we also determine whether the heading error *e* is below the error threshold $\gamma =10^{\circ }$ or not. This allows us to label each time window with class $C_{0}$ or $C_{1}$. We built the dataset by taking *L* observations of $\bar{M}$ over different time windows, here denoted as ${\bar{M}}_{j}$ for j=1,…,L, and the corresponding classes $C_{j}\in \left\{C_{0},C_{1}\right\}$.

For the Tyree Building dataset, we collected L=44 and L=42 samples, respectively, from the corridors LG and G, as shown in column 4 of Table 1. For each experiment, the resulting dataset is a matrix that the *j*-th row is $\left[{\bar{M}}_{j},C_{j}\right]$. We utilise a 10-fold cross-validation to split the datasets into training and test sets, and evaluate the classification performance. The following classification algorithms are evaluated using the software Weka [56]: Support Vector Machine, Multi Layer Perceptron, Decision Tree, 3-Nearest Neighbour, Logistic Regression and Naïve Bayes. The accuracy of the classification is chosen as the performance metric and the average accuracy is reported in Table 3 for each classifier. The numerical results show that Multi Layer Perceptron achieves an average accuracy of 94.82% and outperforms all the other classifiers. We thus select this classifier for the MPD component of CML-PDR.

As we mentioned earlier, for each experiment, we used cross validation to evaluate the performance of the classifiers. Therefore, for each experiment, we first randomly splitted the data into training and test sets. Then the model is learnt from the training data of the experiment and exploited to classify test data from the same experiment. This has been repeated 10 times and the average of accuracy is reported as the classification accuracy.

In our previous study [49], we have attempted to build a model from data of experiments in one corridor at a building and test it on a different corridor in the same or different building. The numerical results show that the classification accuracy highly decreases and it is necessary to train a local model for each corridor in advance. This is due to the spatial diversity of magnetic perturbations and indicates that it is not possible to generalize a unique model for all the environments. Therefore, for each building, it is necessary to train magnetometer measurements for MPD in advance. As this data collection at each building is highly localized, different classifiers should be tested with several thresholds of γ, as hyper parameter of the model, to identify the optimum γ and best classifier for each building.

## 5. Pedestrian Clustering (PC)

The PC component uses WiFi-Direct RSS data to determine whether pedestrians are walking in the same direction. WiFi-Direct [23] is a variation of the traditional WiFi standard, which allows two or more users to directly connect each other without using any intermediate access point. By means of this technology, one phone operates as a server, acting as an access point, while the other phones as clients. As a result, the client phones can collect information, e.g., RSS samples, related to the server phone, by simply performing a WiFi scan.

**Figure 6:**
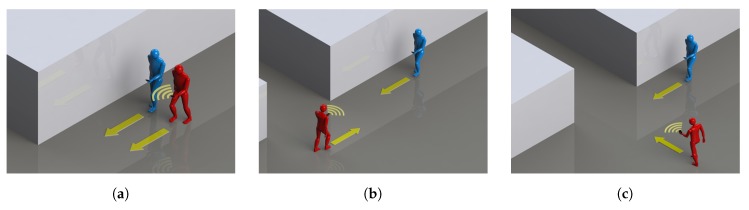
Walking topologies. (**a**) Same heading path, (**b**) Opposite heading path, (**c**) Perpendicular heading path.

To investigate whether WiFi-Direct RSS data can be useful to detect pedestrians walking in the same direction, we carried out a set of experiments as a proof of concept in three different buildings at the UNSW and at the Australian Technology Park (ATP) in Sydney. For each building, we asked two volunteers to walk in the same, opposite, and perpendicular directions as shown in Figure 6. Each pedestrian carried a Samsung Galaxy SIII phone. One of them was configured as a server, transmitting beacons every 0.3 s, and the other as a client.

**Figure 7:**
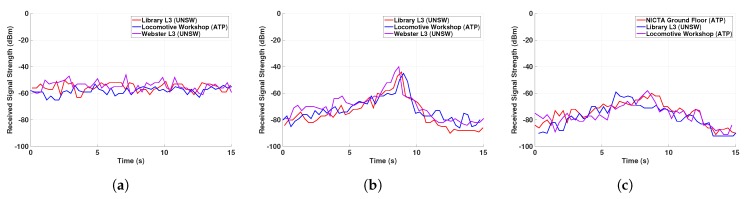
RSS values over time for the three different walking topologies. (**a**) Same heading path, (**b**) Opposite heading path, (**c**) Perpendicular heading path.

Figure 7 plots the RSS measured by the client phone over time for all the experiments, i.e., for the three different buildings and the three different walking topologies. As expected, the RSS is relatively constant for pedestrians walking in the same direction, whereas it varies significantly in the other two configurations with opposite or perpendicular walking directions. This is clearly due to the varying distance between the pedestrians and the related path-loss fluctuations when they are not walking in the same direction. These experiments confirm that a sequence of WiFi-Direct RSS values contains relevant information to infer whether pedestrians are walking in the same direction.

We proposed several time-domain statistical features for RSS measurements and employed the Principal Component Analysis (PCA) to identify the most informative features. After an extensive training and testing, we selected the following five features:

- Rate of change (RoC) of RSS values over two consequent time windows.
- Slope of the line of best fit (SoP) of RSS values in a given time window.
- Root mean square (RMS) of the RSS values in a given time window.
- Minimum (Min) RSS value recorded in a given time window.
- Maximum (Max) RSS value recorded in a given time window.

**Figure 8:**
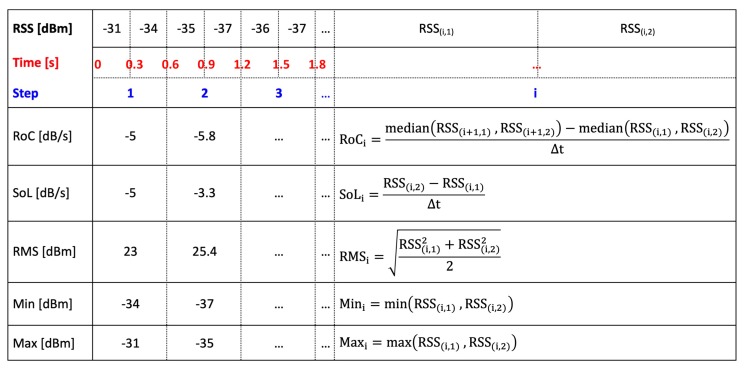
The RSS measurements over the time have been shown on top of the figure. Each step time is 0.6 s and contains two RSS data. For the first two steps, we calculated the proposed features and display them. The RoC for the first step/window is considered equal to the SoP of the first step/window.

To calculate these features, we use again a window size of T=0.6 s, which is roughly the step time and it is twice the beacons transmission time (0.3 s). In Figure 8, we show the time window of RSS measurements and how the features have been calculated.

For the analysis of the PC performance in a larger group of users, we use the dataset of the Tyree Building Scenario in Section 3, which involved four pedestrians walking in the same or opposite directions. One of the pedestrian’s phone was configured as server, while the other three users used RSS data to detect if they were walking in the same direction.

Using Weka, we evaluated the features listed above with six different classifiers and utilising 10-fold cross validation. From the accuracy of the classifiers reported in Table 4, we find that most classifiers achieve an accuracy close to 90%, while Decision Tree performing slightly above the others. Therefore, we use this algorithm to implement the PC component of CML-PDR system. This accuracy highlights that the sampling rate of 0.3 s, combined with the selected features, is efficient. In [57], we have used larger time window that contains more RSS data points to approximate features more precisely. The results show that the performance of PC slightly increases, however the best average accuracy of CML-PDR is achieved for RSS sampling rate of 0.3 s and window size of 0.6 s.

## 6. Distributed Data Fusion (DDF)

In the DDF, heading estimates from multiple people walking in the same direction—filtered by MPD and PC—are aggregated to provide a more accurate estimate of the common heading. This purpose is accomplished by averaging the estimates, so as to take into account the different degree of reliability of measurements. As we mentioned earlier, the rationale of this approach is based on the observation that magnetic perturbations are highly localized in space. Thus, if a number of users are moving with the same heading, it is likely that only a subset of their sensor measurements is corrupted.

The heading estimate ${\bar{h}}_{i,j}$, obtained by user i=1,…,N and in time window j=1,…,L is modeled as: ${\bar{h}}_{i,j}=h_{\mathrm{true}}+w_{i,j}$, where $h_{\mathrm{true}}$ is the true heading and $w_{i,j}$ is the measurement error with variance $\sigma_{i,j}^{2}$.

Cartesian components $\left[{\bar{x}}_{i,j},{\bar{y}}_{i,j}\right]=\left[cos\left({\bar{h}}_{i,j}\right),sin\left({\bar{h}}_{i,j}\right)\right]$ are employed in the combination rather than the headings, so as to avoid ambiguity problems in averaging angle measurements. In the following, only the procedure to draw the global estimate $\hat{x}$ from $\left\{{\bar{x}}_{i,j}\right\}$ will be described, an analogous mechanism is applied to assess $\hat{y}$ from $\left\{{\bar{y}}_{i,j}\right\}$.

Fusion is carried out according to the Best Linear Unbiased Estimator (BLUE) [58]:

(3) $$\hat{x}=\frac{\sum_{i=1}^{N}\sum_{j=1}^{L}{\bar{x}}_{i,j}/\sigma_{i,j}^{2}}{\sum_{k=1}^{N}\sum_{l=1}^{L}1/\sigma_{k,l}^{2}}.$$

We accomplish the above fusion in a distributed way as a cascade of two steps, *fusion over time* and *fusion between users*.

At first, each user performs a data fusion over time by performing a weighted average of the measurements he collected in *L* different time windows as

(4) $${\hat{x}}_{i}=\frac{\sum_{j=1}^{L}{\bar{x}}_{i,j}/\sigma_{i,j}^{2}}{\sum_{k=1}^{L}1/\sigma_{i,k}^{2}}.$$

Note that weighting by the variance inverse allows to account for the different degree of reliability at different windows. For practical implementation, the variances $\sigma_{i,j}^{2}$ are computed by users as sample variances from the measurements collected in window *j*.

Time averaging is then followed by a data fusion between users, which consists in merging the estimates (4) collected by *N* different users,

(5) $$\hat{x}=\sum_{i=1}^{N}\alpha_{i}{\hat{x}}_{i},$$

by a linear combination with weights ${\left\{\alpha_{i}\right\}}_{i=1}^{N}$. In order to guarantee the equality with (3), the weights must be set to $\alpha_{i}=\alpha_{i}^{\mathrm{opt}}≜\sigma_{i}^{-2}/\left(\sum_{k}\sigma_{k}^{-2}\right)$, where $\sigma_{i}^{2}=$var$\left({\hat{x}}_{i}\right)=1/\sum_{j}\sigma_{i,j}^{-2}$. We will consider both $\alpha_{i}=\alpha_{i}^{\mathrm{opt}}$ and the simple average solution using $\alpha_{i}=1$.

A main issue is the distributed implementation of (5). We recall that our aim here is to develop an infrastructure-free solution that can be exploited in all kinds of scenario: a centralized data fusion (CDF) approach is not suited as it relies on a central unit that might be costly or even not available in certain environments. The recent trend in distributed system enables convergence to the centralized global estimate in a fully decentralized way even in large networks with moderate connectivity. Thereby, we propose to implement the fusion (5) by a distributed approach based on consensus [46]; this is an iterative process that consists on estimate exchanges on a peer-to-peer basis among the users of a network in order to reach a consensus for the evaluation of a certain quantity of interest [59]. The network in our scenario is the set of users that are closely located and filtered by MPD and grouped by PC. We can model the network as a graph G=(V,E) where the nodes V={1,…,N} are the users and the edges E⊆V×V are the communication links among the nodes. Let $A=[a_{ij}]$ be the N×N symmetric adjacency matrix, with $a_{ij}=1$ if (i,j)∈E (i.e., if node *j* communicates with node *i*); $a_{ij}=0$ for any (i,j)∉E. The neighbors of a user *i* are denoted by $N_{i}=\left\{k\in V:\left(i,k\right)\in E\right\}$ and $d_{i}=\left|N_{i}\right|$ is the node degree. The Laplacian matrix of the graph is L=D−A with $D=\mathrm{diag}(d_{1},\cdots ,d_{N})$.

In the consensus approach each node updates at each iteration its local estimate and exchanges it with its neighbors until an agreement is reached. At iteration *n* the estimate at user *i* is updated as follows:

(6) $${\hat{x}}_{i}\left(n+1\right)={\hat{x}}_{i}\left(n\right)+\varepsilon W_{i}\sum_{k\in N_{i}}\left({\hat{x}}_{k}\left(n\right)-{\hat{x}}_{i}\left(n\right)\right),$$

where ε is the step size, selected as $0<\varepsilon <2/\lambda_{max}\left(W^{-1}L\right)$, to guarantee convergence, with $\lambda_{max}\left(\cdot \right)$ denoting the maximum eigenvalue of the argument matrix and $W=\mathrm{diag}(W_{1},\cdots ,W_{N})$ [46].

At the first step, each user utilizes its own estimate, i.e., for the first iteration at i=0, the estimates are initialized as ${\hat{x}}_{i}\left(0\right)={\hat{x}}_{i}$. According to [46], if weights $W_{i}$ are chosen as $W_{i}=\sigma_{i}^{2}$ consensus converges to the global BLUE solution in (3) and ${\hat{x}}_{i}\left(\infty \right)=\hat{x}$. On the other hand if $W_{i}=1$, consensus converges to (5) with $\alpha_{i}=1/N$, i.e., to the average of the estimates between different users: ${\hat{x}}_{i}\left(\infty \right)=\frac{1}{N}\sum_{i=1}^{N}{\hat{x}}_{i}$. The two approaches are referred to as *weighted average consensus* (WAC) and *average consensus* (AC), respectively and shown by DDF-W and DDF in the evaluation section.

At the end of consensus processing the users that have taken part in the distributed algorithm spread the final estimate to the users that have been excluded by MPD. In such a way we can take advantage from spatial diversity, as we need just one user with uncorrupted estimate to share the data with the other users.

In the worst case scenario where all users do not overcome the MPD (i.e., they all experienced perturbed measurements), users can employ their previous estimate as the current heading until at least one of them is able to overcome the MPD and provide a new heading estimate for the group. If this scenario happens in the first time instant of the algorithm the users fuse their estimates even if they are all corrupted.

## 7. System Evaluation

The performance of CML-PDR is evaluated in terms of heading and location accuracy in the following, by using the dataset of the real indoor scenarios presented in Section 3. We verify the performance of CML-PDR for different configurations of MPD, PC and DDF algorithms, and compare it to the conventional PDR. We consider the following methods for our analysis:

- Conventional PDR: only magnetometer readings are used to compute the heading without any further processing.
- MPD: only perturbation filtering is used and the simple average is carried out on the filtered heading.
- DDF: only distributed fusion is performed using the AC algorithm.
- DDF-W: only distributed fusion is applied using WAC.
- MPD & DDF: cascade of MPD and DDF.
- MPD & DDF-W: cascade of MPD and DDF-W.
- MPD & CDF: the centralized unweighted computation of (5) is employed after the MPD.
- MPD & CDF-W: the centralized weighted computation of (5) is employed after the MPD.

### 7.1. Localization Performance Analysis

To validate CML-PDR, we utilize the experiments of the described in Section 3.1. We first exclude topology B, as in this topology none of the pedestrians walked in the same direction of U4, which worked as a server, and as a consequence, the remaining users miss the opportunity to cooperate.

Table 5 presents the average heading errors of the pedestrians for the eight experiments indexed in the first column according to Table 1. The second column shows the average heading errors obtained from conventional PDR based on raw magnetometer readings only, without using any component of CML-PDR. In columns 3–6 we show the heading accuracy obtained by the cooperative approach where PC is exploited to detect all the pedestrians walking in the same direction. The results in brackets refer to the ideal case of perfect clustering of users by the PC component. In Columns 3 and 4, we show the average heading errors when DDF-W and DDF are employed respectively without MPD filtering. The former exploits $W=\sigma^{2}$ and the latter use w=1 as the weight. Finally, the performance of the combined MPD and DDF algorithms are shown in the last two columns.

Compared to the conventional PDR, we can notice that the proposed cooperative method significantly reduces the average heading error. The lowest error is achieved by the combination of MPD and DDF with an average value of 4.55${}^{\circ }$ (2.97${}^{\circ }$ in the ideal scenario) and on average reduces the heading estimation error from 32.27${}^{\circ }$ to 4.55${}^{\circ }$ (86% error reduction) compared to the conventional PDR system. It can also be observed that the PC component has a significant impact on the performance of CML-PDR. In fact, in the ideal scenario when PC accuracy is 100%, the average heading error is reduced to 2.97${}^{\circ }$. Although the proposed PC algorithm achieves an accuracy of 90%, the gap in the heading error with the ideal scenario is noticeable (4.55${}^{\circ }$ vs. 2.97${}^{\circ }$).

We examine now the system performance in terms of location accuracy. In the experiments, the subjects were instructed to walk at a constant speed (1 m/s on average) taking constant step lengths (0.6 m approximately). User positions are estimated at each pedestrian step, assuming perfect knowledge of the step length. The position estimate is thus computed every 0.6 s (at each pedestrian step) by combining the heading estimate with the known step length. We evaluate the localization performance in terms of Root Mean Square Error (RMSE) of the location estimate by averaging over different users and paths. In every experiment, the RMSE, over the entire trajectory of M steps, has been computed as:

(7) $$RMSE=\sqrt{\frac{\sum_{i=1}^{M}{\left(x_{i}-x_{i}^{\prime }\right)}^{2}+{\left(y_{i}-y_{i}^{\prime }\right)}^{2}}{M}},$$

where $\left(x_{i},y_{i}\right)$ are the coordinates of the true location and $\left(x_{i}^{\prime },y_{i}^{\prime }\right)$ are the coordinates of the location estimate for the *i*th step.

By means of PC, MPD and DDF our system achieves a RMSE of 1.38 m. This result represents a remarkable improvement of 79% if it is compared to the conventional PDR, which in turn achieves an accuracy of 6.57 m.

In order to verify the robustness of CML-PDR, we evaluated it in a more practical scenario, where users make several turns and do not walk at the same velocity. We repeated the same procedures described in Section 3 for collecting data from three volunteers in a square path in the CSE laboratory at UNSW. Several obstacles were present in this environment to ensure shadowing and multi-path effects on RSS. We asked the pedestrians to walk normally and at their personal walking speed, while maintaining the distance between each other such that they could form a group. Again we utilised $\gamma =10^{\circ }$ for MPD.

**Figure 9:**
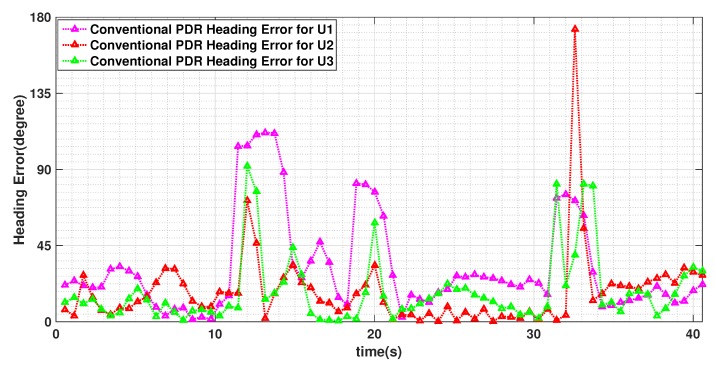
Heading estimates of three users using conventional PDR. The pedestrians walk in different speed and over the square path 3 turns shown in Figure 11.

**Figure 10:**
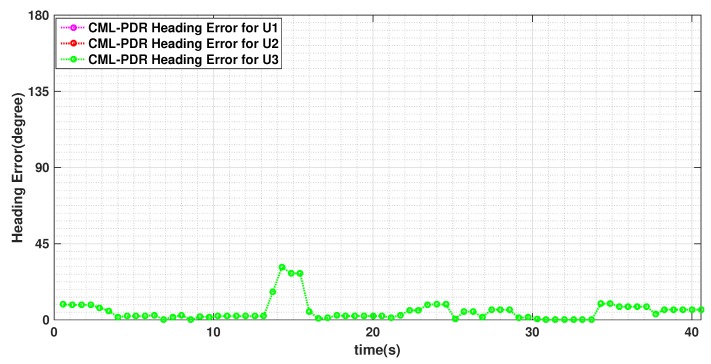
Heading estimates of three users using CML-PDR. The pedestrians walk in different speed and over the square path 3 turns shown in Figure 11. Since all the users collaborate to estimate consensus heading, all three users have the same heading error.

In Figure 9, we plot the estimated headings of the three users for this experiment when convention PDR, i.e., non-cooperative approach, is used. The colored points represent the three pedestrians heading estimates during the experiment. The figure shows that the estimated heading error due to the magnetic perturbation is highly spacial but all the pedestrians follow almost similar patterns at different time. In Figure 10, we show the heading error of these users using CML-PDR. Obviously, all the users have the same heading error that notably decreases.

**Figure 11:**
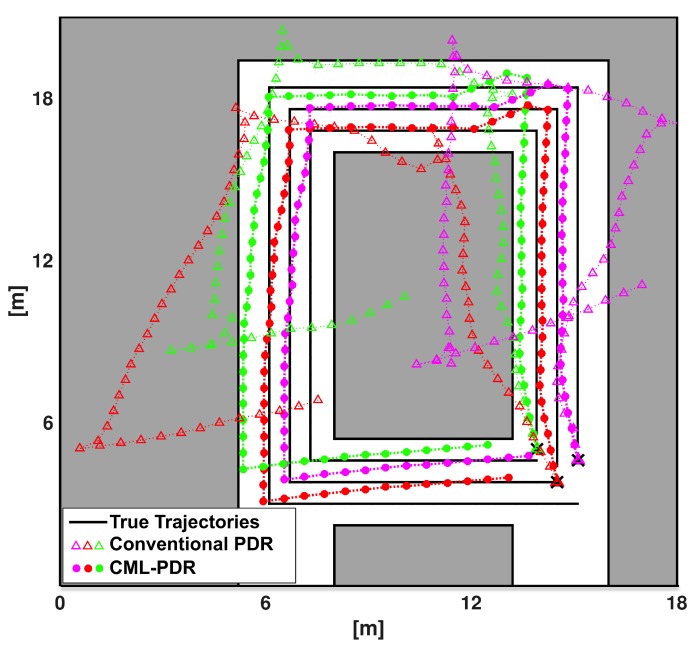
Localization of three users walking in different speed and over a path with several turns to verify the performance of the system for a complex scenario. Colored markers represent position estimates of the three pedestrians, while black lines indicate the true trajectories.

We show the position estimates for both CML-PDR and conventional PDR approaches in Figure 11. It should be noticed that we did not take into account (a-priori) the planimetry of the room in the evaluation. The grey color shows the shared office space where desks and computers are placed and pedestrians could not walk through. Our results show that localization RMSE for CML-PDR is 0.66 m that results in a 81% improvement if compared to the conventional PDR (3.53m). This result validates our proposed method in a more complex scenario and confirms the prominent results obtained in previous experiments.

### 7.2. Localization Performance vs. Degree of Cooperation

In this section, the performances of data fusion algorithms are analyzed in terms of convergence time and degree of cooperation among large group of users. This analysis is necessary because unlike the centralized approach, the performance of the distributed solution depends on the connectivity degree of the network used for sharing. To perform this evaluation, we use the Large-Scale Scenario with 15 and 18 users described in Section 3.2. This dataset allows us to simulate different values of cooperation radius, i.e., the maximum distance at which the users are able to cooperate with each other, and test the performance of cooperative localization versus the degree of cooperation among the users. Note in particular that the scenario herein considered, where a node is enabled to participate to the fusion according to MPD filtering outcome, allows the formation of small fusion groups and the isolation of nodes with non-perturbed estimates. This may generate a performance degradation in the distributed approach with respect to the centralized one, where a unique fusion group is always present regardless of the network topology. Obviously, the distributed solution is expected to perform as the centralized one as the cooperation radius increases.

**Figure 12:**
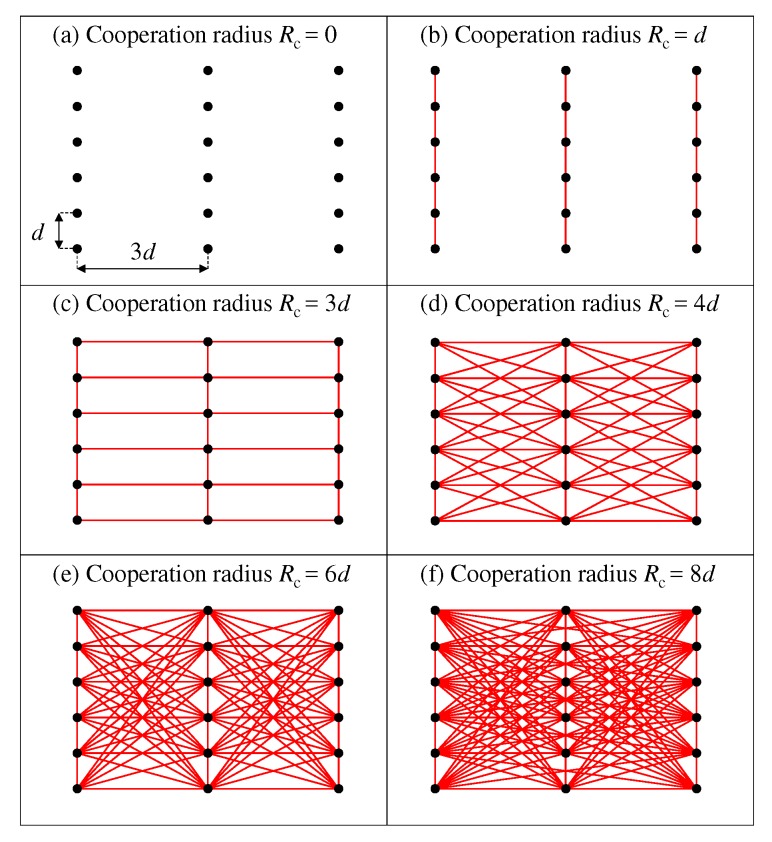
Connectivity graph for cooperation radius $R_{c}\in \left\{0,d,3d,4d,6d,8d\right\}$. Nodes are plotted in black and communication links are shown as red lines.

We examine the effect of the network connectivity by using cooperation radius $R_{c}\in \left\{0,d,3d,4d,6d,8d\right\}$ m with d=0.5 m. The corresponding graph topologies are shown in Figure 12. Note that these levels of connectivity are reached only if the heading estimates at all users have overcome the MPD filtering. In other words, each graph in Figure 12 depicts the maximum cooperation degree for the considered value of $R_{c}$. The case $R_{c}=0$ means no communication among nodes (each user estimate reduces to the non-cooperative assessment (4)), whereas $R_{c}=8d$ corresponds to all-to-all connectivity (i.e., each user reaches the centralized estimate). Intermediate values reproduce network configurations with limited connectivity among nodes.

**Figure 13:**
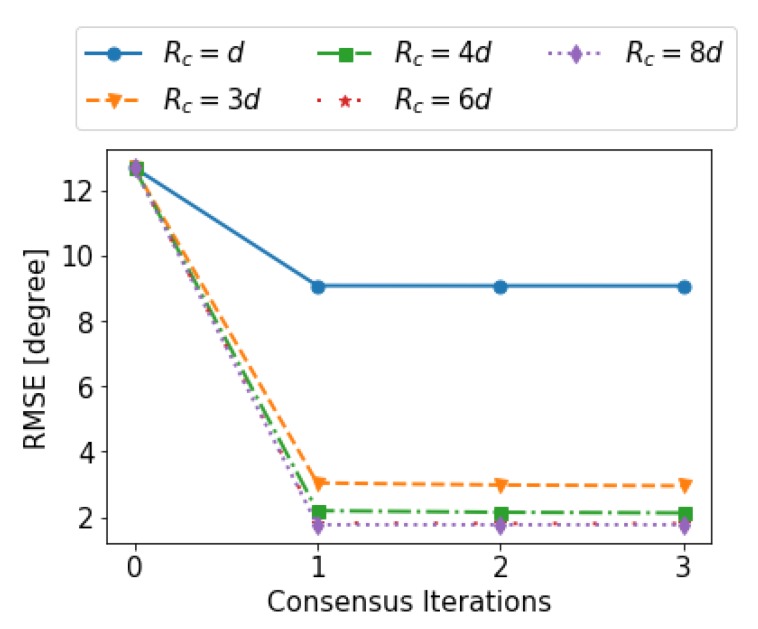
Heading RMSE as a function of the iterations and of the cooperation radius for the distributed approach.

In Figure 13, we show heading RMSE for different iterations of the DDF processing and different values for radius of cooperation. It can be seen that only one iteration and a cooperation radius of $R_{c}=4d=2$ m (red color) is comparable to $R_{c}=8d=4$ m (green color) that is a fully connected network and performs similar to CDF. Therefore, even a small degree of connectivity is enough for the DDF approach to approximate the performance of the CDF. In this instance, MPD & DDF provide heading RMSE of $2.1^{\circ }$ whereas and MPD & CDF achieves the accuracy of $1.7^{\circ }$. Therefore, for Large-Scale scenario and cooperation radius of $R_{c}=4d=2$ m, CML-PDR provides an error reduction of 88% with respect to conventional PDR.

**Figure 14:**
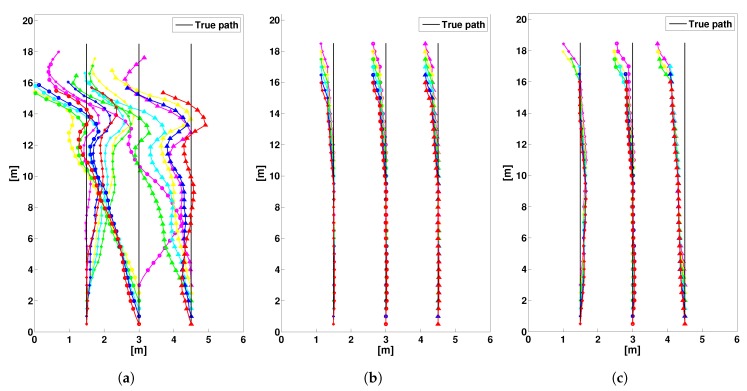
Trajectories of 18 users by cooperative and non cooperative processing. Each marker and color combination uniquely represents a user. Black lines indicate the true trajectories. Each trajectory is covered by a group of 6 users that are walking vertically aligned, as indicated in Figure 12. The estimates are represented by colored markers, with a different color for each user and a different marker for each of the 3 trajectories. (**a**) Conventional PDR; (**b**) MPD & CDF; (**c**) MPD & DDF (CML-PDR).

In order to analyze the effect of cooperation radius $R_{c}$ on the localization accuracy, in Figure 14 we have compared the trajectories of the experiment 4 in Large-Scale Scenario for the conventional PDR, MPD & CDF and MPD & DDF (CML-PDR). Obviously, the trajectories estimated by the centralized and distributed approachs in Figure 14b,c achieve noticable localization error reduction compare to the conventional PDR in Figure 14a. For the distributed approach, we use a cooperation radius $R_{c}=4d$ and the the localization error obtained by CML-PDR is 0.18 m that corresponds to the localization error reduction of 93% compared to the conventional PDR. Moreover, despite limited connectivity between users, our method closely matched the trajectories estimated by the centralized approach (MPD & CDF), as can be appreciated in Figure 14b,c.

**Figure 15:**
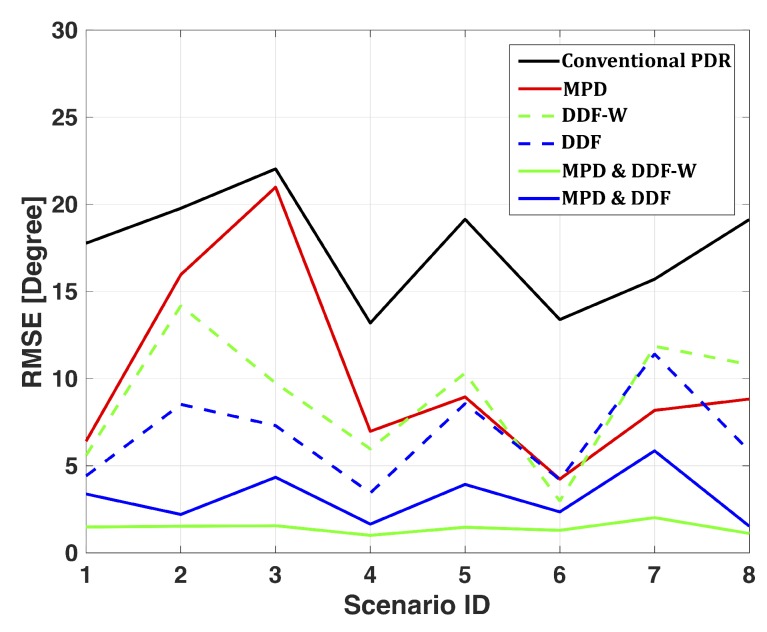
Heading RMSEs for the eight localization scenarios of the Large-Scale Scenario.

As we mentioned earlier, one objective of performance analysis for Large-Scale Scenario is to verify the heading error reduction of our proposed method for a larger group of pedestrians. Similar to Section 7.1, we assess the performance of CML-PDR for the large-scale scenario and compared the heading estimates obtained by the conventional PRD, MPD, data fusion (DDF-W and DDF), and combined MPD with data fusion (MPD & DDF-W and MPD & DDF). In Figure 15, we depict the RMSE of the heading estimate of these approaches for scenario 1 to 8 in Table 2. In this analysis, we assume perfect user clustering (i.e., PC with 100% accuracy). Results in Figure 15 highlight that both MPD and DDF are needed for reliable heading estimation as the error is high when only one component is implemented. The combined approach for large scale scenario yields an error reduction of 91% (MPD & DDF-W) and 80% (MPD & DDF) compared to conventional PDR, with a RMSE of respectively $1.45^{\circ }$ and $3.45^{\circ }$. The localization accuracy obtained using conventional PDR is 2.75 m, while CML-PDR achieves a the location RMSE of 0.55 m, with an improvement of 80% of performance. As we expect, the MPD & DDF-W method overcomes the MPD & DDF since it weights the estimates according to a reliability metric. However, depending on the selected weights, the performance of the MPD & DDF-W could be better or worse than MPD & DDF. It is due to the fact that the MPD & DDF-W performance relates to the measurement bias that occurs in the scenarios and it is not accounted for in the weighting of BLUE (which assumes unbiased observations). A possible way to deal with this is performing first DDF and then DDF-W using as reference for computing the weights. We have discussed this issue in our previous work [49].

## 8. Conclusions and Future Works

The main purpose of this paper is to overcome one of the main limits of PDR indoor localization systems: heading estimation. This work provides an accurate and efficient method to assess the direction of motion of pedestrians relying only on magnetometers embedded in smart devices and peer-to-peer connectivity. We evaluated the performance of our approach using real experiments in indoor environments and the performance exhibits a significant heading error reduction (86%) with respect to the conventional PDR, corresponding to an average heading error of $4.55^{\circ }$. This allows to achieve a location accuracy of 1.38 m, which represents 79% error reduction with respect to conventional PDR approach.

CML-PDR relies on the spatial diversity of magnetic perturbation inside a building and it is necessary to train magnetometer measurements for MPD in advance. Comparing to fingerprinting database, the data collection for this training does not require exhaustive labour work and substantial cost, however the magnetometer data training is needed for every building. This is one of the limitation of CML-PDR. Another disadvantage of this method is that the initial position should be shared with the smartphone to create the trajectory. This is due to the fact that PDR estimates the location at each step and based on the previous location. Therefore it is necessary to provide the initial position at the entrance of the building with the device.

In our evaluations, we show that the performance of CML-PDR is robust in a complex environment with several turns and a long distance (60 m) while the pedestrian holds the smartphone horizontally. Future work will address real-time testing in large indoor spaces, such as airport and museum, by considering more complicated paths, longer distances and as a crowded environment. We also verify the performance of CML-PDR when the pedestrian holds the smartphone at different position and focus on the enhancement of the PC component to further improve the localization accuracy. Other D2D communication, such as Bluetooth iBeacon [60] and LTE proximal discovery beacons [61], will be considered as emerging technologies to compare the power consumption CML-PDR and investigate the coverage and effect of multi path in an open area.

## Figures and Tables

**Table 1:** Small-Scale Scenario Experiments.

Experiment ID	Corridor	Topology	Number of Steps (Length)	True Heading			
				U1	U2	U3	U4
1	LG	A	44 (26.4 m)	99.18${}^{\circ }$	99.18${}^{\circ }$	99.18${}^{\circ }$	99.18${}^{\circ }$
2			44 (26.4 m)	279.18${}^{\circ }$	279.18${}^{\circ }$	279.18${}^{\circ }$	279.18${}^{\circ }$
3		B	44 (26.4 m)	99.18${}^{\circ }$	99.18${}^{\circ }$	99.18${}^{\circ }$	279.18${}^{\circ }$
4			44 (26.4 m)	279.18${}^{\circ }$	279.18${}^{\circ }$	279.18${}^{\circ }$	99.18${}^{\circ }$
5			44 (26.4 m)	99.18${}^{\circ }$	99.18${}^{\circ }$	99.18${}^{\circ }$	279.18${}^{\circ }$
6			44 (26.4 m)	279.18${}^{\circ }$	279.18${}^{\circ }$	279.18${}^{\circ }$	99.18${}^{\circ }$
7	G	A	42 (25.2 m)	99.18${}^{\circ }$	99.18${}^{\circ }$	99.18${}^{\circ }$	99.18${}^{\circ }$
8			42 (25.2 m)	279.18${}^{\circ }$	279.18${}^{\circ }$	279.18${}^{\circ }$	279.18${}^{\circ }$
9		C	42 (25.2 m)	99.18${}^{\circ }$	279.18${}^{\circ }$	279.18${}^{\circ }$	279.18${}^{\circ }$
10			42 (25.2 m)	279.18${}^{\circ }$	99.18${}^{\circ }$	99.18${}^{\circ }$	99.18${}^{\circ }$
11			42 (25.2 m)	99.18${}^{\circ }$	279.18${}^{\circ }$	279.18${}^{\circ }$	279.18${}^{\circ }$
12			42 (25.2 m)	279.18${}^{\circ }$	99.18${}^{\circ }$	99.18${}^{\circ }$	99.18${}^{\circ }$

**Table 2:** Large-Scale Scenario Experiments.

Scenario ID	UNSW Building	True Heading	N${}^{\circ }$ of Users	N${}^{\circ }$ of Experiments
1	Library, 3rd Floor	188.98${}^{\circ }$	3	6
2	Old Main Building, Ground Floor	279.23${}^{\circ }$	3	6
3	Old Main Building, Ground Floor	99.46${}^{\circ }$	3	6
4	Robert Webster Building, LG Floor	99.26${}^{\circ }$	3	6
5	Robert Webster Building, LG Floor	279.18${}^{\circ }$	3	5
6	ABS Building, 1st Floor	99.26${}^{\circ }$	3	6
7	ABS Building, 1st Floor	279.15${}^{\circ }$	3	6
8	Electrical Engineering Building, 2nd Floor	98.90${}^{\circ }$	3	6

**Table 3:** MPD Performance for Different Classifiers.

Accuracy (%)					
Support Vector Machine	Multi-Layer Perceptron	Decision Tree	K-Nearest Neighbour (K=3)	Logistic Regression	Naïve Bayes
77.28	94.82	94.21	92.32	78.29	82.15

**Table 4:** Comparison of classifiers accuracy for PC.

Accuracy (%)					
Support Vector Machine	Multi-Layer Perceptron	Decision Tree	K-Nearest Neighbour (K=2)	Logistic Regression	Naïve Bayes
90.37	90.66	90.90	90.17	90.37	88.83

**Table 5:** Heading errors in degree. Data in parenthesis represent outcomes obtained by PC performing with 100% accuracy.

Experiment ID	Conventional PDR (${}^{\circ }$)	Cooperative (${}^{\circ }$)			
		DDF-W	DDF	MPD & DDF-W	MPD & DDF
1	57	7.98 (6.19)	16.78 (15.07)	8.77 (8.03)	4.73 (3.84)
2	18.73	12.23 (11.7)	5.03 (3.93)	5.19 (4.94)	2.03 (1.39)
7	46.59	13.24 (4.11)	26.59 (18.95)	13.04 (6.61)	9.75 (2.01)
8	14.21	0.67 (0.55)	7.24 (7.23)	0.82 (0.81)	1.83 (1.85)
9	7.71	2.49 (1.68)	5.56 (1.95)	1.78 (1.78)	1.61 (1.61)
10	53	30.15 (25.97)	51.44 (37.07)	8.84 (5.76)	8.84 (5.75)
11	9.33	1.64 (1.79)	3.15 (1.82)	1.77 (1.77)	1.77 (1.77)
12	51.58	23.06 (17.28)	40.70 (25.84)	5.54 (5.01)	5.85 (5.53)
**Average**	**32.27**	**11.42 (8.66)**	**19.56 (13.98)**	**5.72 (4.33)**	**4.55 (2.97)**
